# Radical resection of a giant cell tumor of the distal ulna and reconstruction with a 3D-printed distal ulnar prosthesis: A case report

**DOI:** 10.1097/MD.0000000000043504

**Published:** 2025-07-18

**Authors:** Lifu Wang, Chuanle Yang, Zhixiang Gao, Shaoyun Zhang, Xiaofeng Zheng, Lijuan Liu, Cong Xiao

**Affiliations:** a Department of Orthopedics, The Third Hospital of Mianyang·Sichuan Mental Health Center, Mianyang, China; b Department of Nursing, The Third Hospital of Mianyang·Sichuan Mental Health Center, Mianyang, China.

**Keywords:** 3D printing, distal ulna, giant cell tumor, personalized prosthesis

## Abstract

**Rationale:**

Giant cell tumors (GCTs) of the distal ulna are rare. Despite their benign nature, these tumors can exhibit local aggressiveness and have the potential to recur after undergoing conventional treatment involving curettage and bone grafting. For aggressive GCTs, radical resection is more suitable. However, these methods have inherent shortcomings, including a high postoperative recurrence rate and poor mechanical stability. Medical applications of the 3D printing technique are on the rise, with orthopedic repair and reconstruction benefiting from the successful implementation of custom-made prostheses created via 3D printing technology.

**Patient concerns:**

A 38-year-old male patient presented to a local clinic complaining of a slow-growing painless on over the ulnar aspect of his left wrist for 1 month. The biopsy confirmed the diagnosis of GCTs and was graded as Campanacci grade III.

**Diagnoses:**

The diagnosis of GCTs of the distal ulnar was confirmed by biopsy.

**Interventions:**

The patient received a 2-month preoperative adjuvant chemotherapy with denosumab. Then, a radical distal ulnar resection and hemiarthroplasty are performed using a 3D-printed prosthesis to reconstruct wrist joint function.

**Outcomes:**

During the 2-year follow-up after the surgery, there were no signs of recurrence, and the left wrist function of the patient remained normal.

**Lessons:**

A well-fitted personalized prosthesis, made using 3D printing techniques, can effectively reconstruct joint function and bone defects resulting from radical distal ulnar resection due to GCTs. This case demonstrates that the combination of radical resection of a GCT in the distal ulna and reconstruction with a 3D printed personalized prosthesis could lead to successful oncologic and functional outcomes.

## 1. Background

Giant cell tumors (GCTs) are benign but locally aggressive tumors, usually occurring at the epiphyses of the distal femur, proximal tibia, and distal radius. GCTs occurring in the upper extremity are not commonly observed, comprising only about 3% to 5% of all primary bone tumors.^[1,2]^ GCTs of the distal ulna are extremely rare.^[3]^ The highest incidence rate is observed around the age of 30, with approximately 70% of individuals being diagnosed between their twenties and forties. The treatment approach for GCTs located in the distal ulna follows conventional methods, which involve curettage and bone grafting for less active types and extensive resection for more aggressive lesions. However, the functional implications of wide excision of the distal ulna should be considered, as it affects forearm rotation and plays a crucial role in bearing loads.^[4,5]^ Currently, the accepted GCTs of the distal ulna involves performing wide local excision, with or without stabilizing the proximal end of the ulna. While previous research has demonstrated promising outcomes solely through the excision of the distal ulna,^[3,6]^ distal ulna resections without reconstruction resulting instability of the ulna stump.^[7]^ A recent meta-analysis of distal radioulnar joint arthroplasties has revealed that total distal radioulnar joint prosthesis has infrequent complications.

The utilization of three-dimensional (3D) printing technique in the clinic field is on the rise, with prostheses created through this technology exhibiting exceptional accuracy and customization. These 3D-printed prostheses have also shown promising clinical outcomes for orthopedic repair and reconstruction.^[8,9]^ However, research on the application of 3D-printed personalized prostheses in distal ulna GCTs resection is scarce. In this study, we present a case of distal ulna GCTs in which satisfactory clinical effects were achieved by reconstructing the radial-ulnar joint using 3D-printed personalized prostheses following distal ulna resection. This anonymous case report was published with the consent of the patient and his family.

## 2. Case presentation

**Figure 1.**
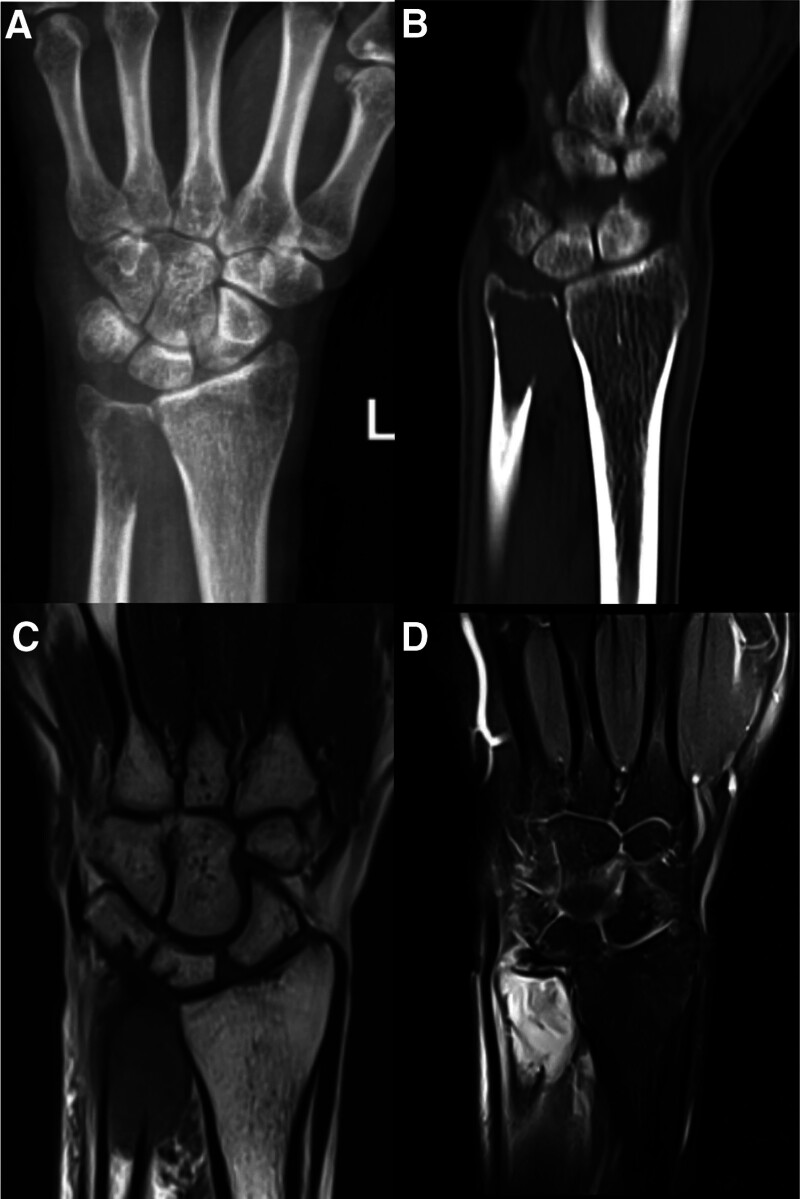
(A) X-ray demonstrated an expansile lytic lesion of the left distal ulna; (B) CT scans demonstrated the lesion has penetrated the cortex; (C) MRI of lesion in left wrist in T1-weighted image; and (D) T2-weighted image view.

A 38-year-old male patient presented to a local clinic complaining of a slow-growing painless on over the ulnar aspect of his left wrist for 1 month, with no apparent trigger. No vascular or neurological symptoms were observed. Laboratory tests showed no significant abnormalities, and the patient denied any history of trauma or other diseases. Radiographs revealed an expansile lytic lesion of the distal ulna (Fig. 1A), and computed tomography (CT) scan revealed that the lesion has penetrated the cortex (Fig. 1B). Magnetic resonance imaging detected a lesion measured 4 cm in length and 2 cm in width, expanding and partially destroying the cortex and presenting low intensity on T1-weighted images and a heterogeneous signal on T2-weight images (Fig. 1C, D). To make a clear diagnosis, he was considered for whole-body fluorodeoxyglucose (FDG) positron emission tomography/CT. FDG positron emission tomography/CT revealed a lesion with intense FDG concentration in the distal ulnar. Then we performed a biopsy and sent the specimen to the department of histopathology (Appendix Figure S1, Supplemental Digital Content, https://links.lww.com/MD/P487). The biopsy confirmed the diagnosis of GCTs and graded as Campanacci grade III. The patient underwent preoperative adjuvant chemotherapy with 5 doses of Denosumab over a 2-month period.

**Figure 2.**
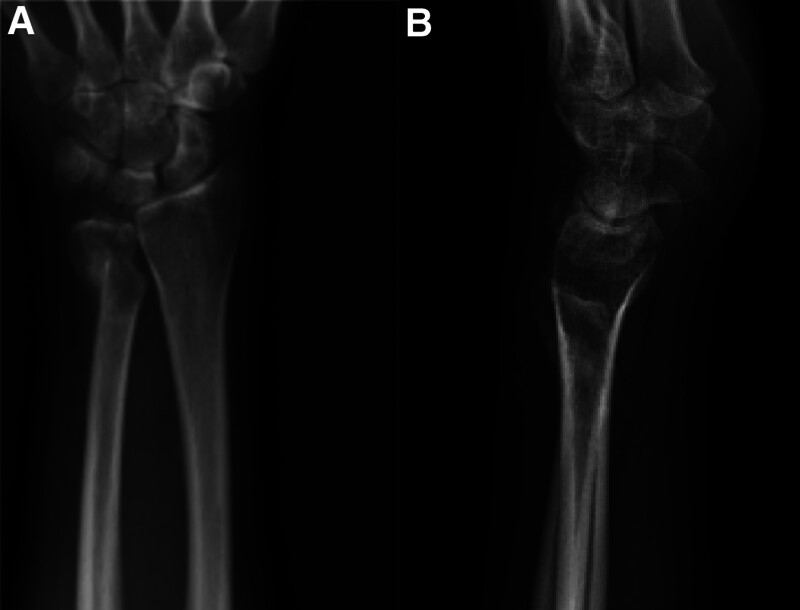
X-ray demonstrated a pathological fracture in the distal ulnar. (A) Anteroposterior view; (B) lateral views.

Four months later, the patient presented again complaining of pain and swelling of the ulnar aspect of his left wrist and radiographs revealed a pathological fracture in the distal ulnar (Fig. 2).

The surgical options were discussed, taking into consideration the patient’s giant cell tumor grade and pathological bone fracture. A radical resection was considered. The patient wanted to continue in his current job, which involved good function of wrist. Considering the above factors, we have ultimately decided on a surgical plan of radical distal ulnar resection and hemi-arthroplasty using a 3D-printed prosthesis to reconstruct wrist joint function.

### 2.1. Prosthesis design

**Figure 3.**
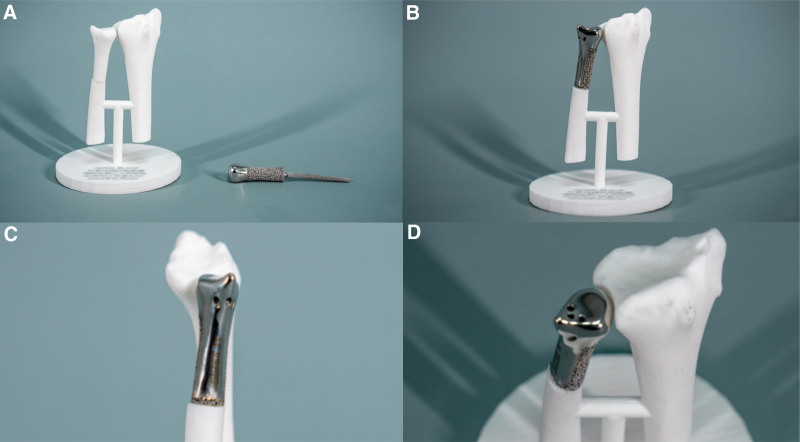
A life-sized sterilizable plastic 3D-printed model of the left distal ulna and radius with the 3D-printed prosthesis (A–D).

The prosthesis was designed by a team of surgeons, and the design procedures are demonstrated as follows. First, the lesion site, including the radius and wrist joint of the affected hand, was scanned using a 128-row spiral CT. By importing the CT data into Mimics software (version 17.0; Materialise NV, Belgium), a 3D model of the distal ulna that included the tumor was established to determine the line for osteotomy. Subsequently, the prosthesis was simulated and designed based on the actual defect and anatomy of the distal ulna. The designed prosthesis has smooth contact surfaces with the radius, as well as with the carpal bones. In addition, 3 holes are designed at the distal end of the prosthesis to sutured and fix triangular fibrocartilage complex. Finally, the prosthesis was printed using titanium alloy powder with a 3D printer and was sterilized by autoclaving before use (Fig. 3). At the same time, 3D prototypes of prosthetics are also printed simultaneously, but using polyethylene as the material.

### 2.2. Surgery procedure

**Figure 4.**
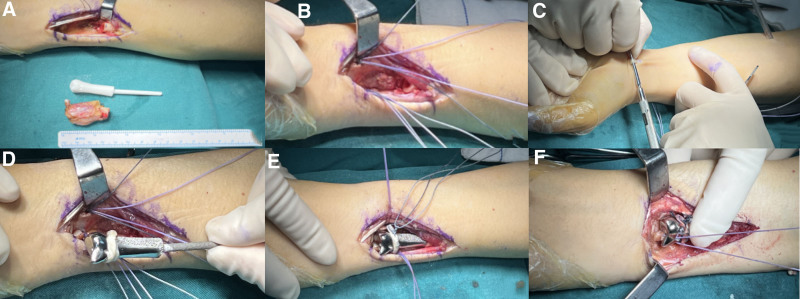
Surgery procedures. (A) The lesion was excised as a whole; (B) suture anchors were inserted in radius; (C) palmaris longus tendon was harvested; (D) the prosthesis was wrapped by palmaris longus tendon and sutured to the anchors on the radius; (E) installed the prosthesis; (F) repair the triangular fibrocartilage complex (TFCC).

A preoperative magnetic resonance imaging and CT scan were performed to determine the proximal margin of resection which was determined to be 3.5 cm proximal to the ulnar corner of the articular surface. The surgical approach is located on the back side. The tumor was approached through an I-shaped incision with a longitudinal component over the distal ulna. A wide extracapsular resection was performed to not violate the tumor capsule. The dorsal sensory branch of the radial nerve and the extensor carpi ulnaris tendon, both structures are dissected and protected around the tumor (Fig. 4A). After the specimen was extracted as a whole, utmost care was taken to ensure optimal control of bleeding. The wound was irrigated and then the reconstructive procedure began.

In the radius bone of the distal radioulnar joint, two 3.0 mm suture anchors were inserted, along with spare anchor sutures (Fig. 4B). A 4 cm portion of the palmaris longus tendon was harvested (Fig. 4C). The palmaris longus tendon was wrapped around the prosthesis, pulled tightly, and then sutured to the anchors on the radius. Finally, the 3D-printed prosthesis was installed (Fig. 4D, E). The triangular fibrocartilage complex was repaired and sutured to the prothesis (Fig. 4F).

**Figure 5.**
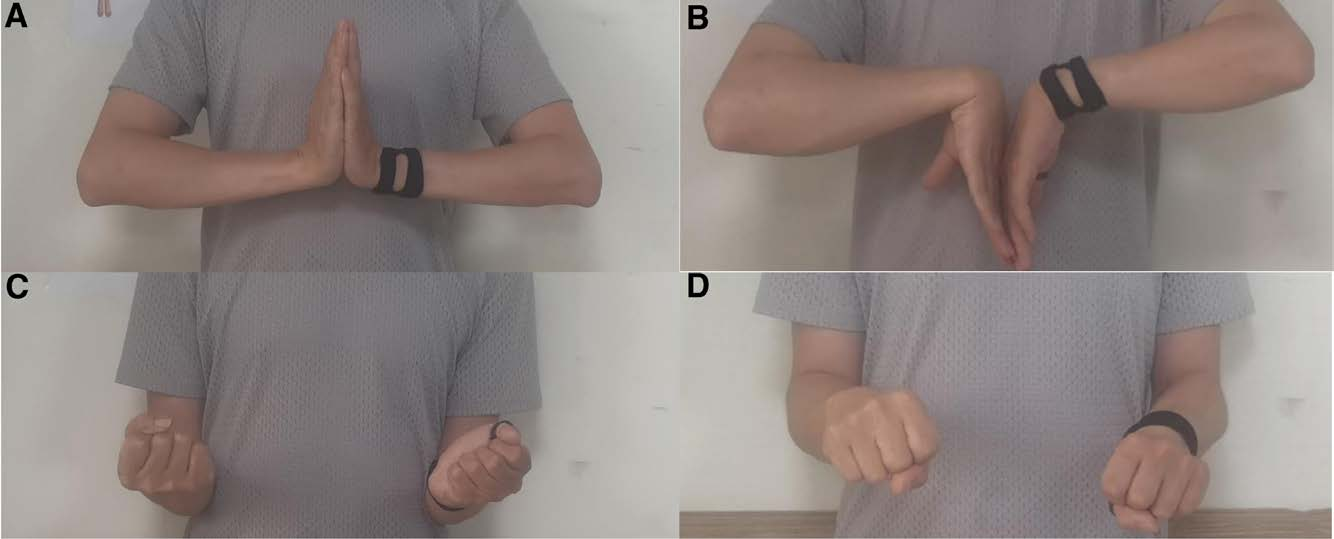
Range of motion at the 1-year follow-up (A–D).

**Figure 6.**
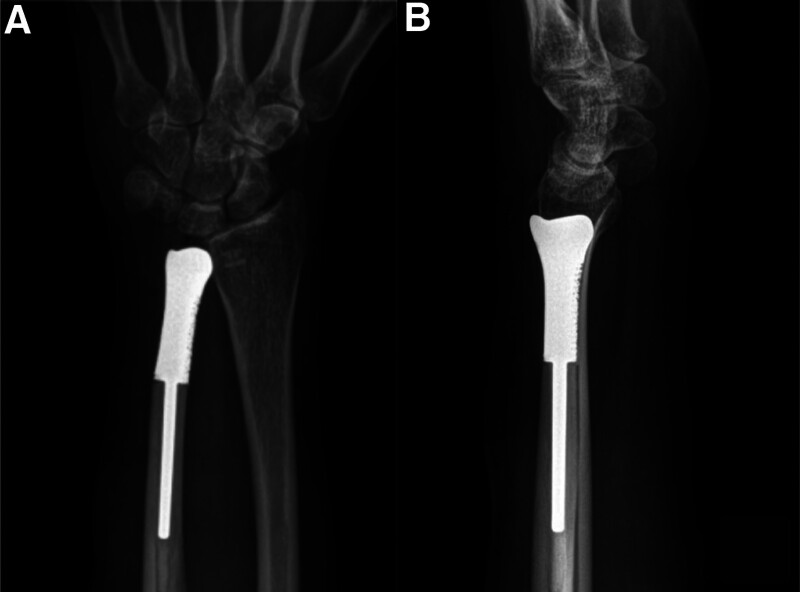
Postoperative radiographs at 1-year follow-up.

Full pronation and supination of the forearm and stability of the distal radioulnar prosthesis was confirmed intraoperatively. The extensor retinaculum was repaired with 4-0 nylon mattress sutures and the skin closed with interrupted 4-0 nylon mattress sutures. The forearm was immobilized in neutral position in a sugar-tong splint and the patient was admitted for 24 hours for elevation and intravenous antibiotics. The sugar-tong splint was removed 10 days postoperatively to reveal uneventful healing of the incision and sutures were removed. The patient began pronation and supination exercises under the direction of a hand therapist. Permanent histology confirmed negative margins both for bone and soft tissue. The wristlet was discontinued 2 months postoperatively. He had full supination, pronation, flexion, and extension 1 year postoperatively (Fig. 5). The radiographic indicated that the prothesis fitted well with the ulna and no signs of loosening or other complications were found (Fig. 6). No intra- or postoperative complications occurred.

## 3. Discussion

GCTs of the distal radius are extremely rare, with a reported incidence rate ranging from 0.5% to 3% among all GCTs.^[10–12]^ In a large-scale study including 3222 cases of GCTs, the cumulative incidence rate of GCTs in the distal radius was found to be 1.27% (47 cases).^[13]^ Different surgical approaches have been suggested for managing GCTs of the distal ulna, such as sole curettage,^[14]^ curettage combined with bone grafting, cryotherapy, application of polymethyl-methacrylate or phenol, radiation therapy, solitary resection,^[15]^ and resection accompanied by an allograft or autograft.^[14]^

Although GCTs is classified as a nonmalignant tumor, it exhibits locally aggressive behavior and carries a notable likelihood of recurrence, especially in cases where the lesion is only curetted or even when adjuvant therapy is employed. One study found that the recurrence rate after curettage ranged was 28.5%,^[16]^ while study reported a decrease in recurrence from 61% to 22% when cement, instead of bone graft, was used to fill the surgical void.^[17]^ The recurrence rate of GCTs is approximately 25% among patients, while curettage has been linked to success rates of up to 50%.^[1]^

Due to the majority of GCTs patients being young adults and the high recurrence rate of GCTs, it is crucial to maximize joint salvage to optimize long-term functionality. Therefore, in our case, curettage either alone or with adjuvant therapy is no longer appropriate because of the Campanacci grade III. Potential surgery option encompassed either performing a radical resection solely on the ulnar distal or combining it with reconstruction techniques. Some authors argue that there is no universal consensus on the necessity of reconstruction after removing the distal ulna for tumors.^[6,18,19]^

Opposition viewpoints suggest that weakness, impingement of the stump, and translation of the ulnocarpal joint may potentially occur following a simple excision procedure, thus indicating the need for reconstruction techniques.^[20–22]^ A biomechanical study suggested that a simple distal ulnar resection could lead to the production of radioulnar convergence and increased anteroposterior translations.^[23]^ These were consistent with another published study.^[24]^ Pronator teres and biceps muscles cause dorsal displacement of the radius during pronation and palmar displacement during supination. Ulnar head arthroplasty and soft tissue repair prevent radioulnar convergence and decrease anterior posterior translation of the distal radioulnar joint. Due to the occurrence of these tumors in young patients with high demand, performing only distal ulnar resection surgery may not provide sufficient strength and functionality.

Currently, nonconstrained hemi-arthroplasty of the distal ulna has been described for reconstruction of the distal ulna after resection of GCTs.^[25,26]^ In addition, 2 case reports also described the use of Scheker constrained distal radioulnar joint arthroplasty after resection of giant cell tumor in the distal end of the radius.^[7,27]^

However, conventionally configured prostheses for distal ulna GCTs are currently limited due to the low incidence of such tumors, and studies on the use of prostheses for the reconstruction in patients with distal ulna GCTs have been limited to a handful of case reports. The application of 3D printing technologies in the field of orthopedics has provided novel methods of bone defect repair and reconstruction following resection of distal ulna GCTs. The perfect fit between the prosthesis and the defect site minimizes the possibility of postoperative recurrence and facilitates maintenance of the wrist’s mechanical stability.

In the present study, 3D-printed personalized prostheses were used for reconstruction after tumor resection in young patient with distal ulna GCTs. Postoperative X-ray examination results revealed a satisfactory fit of the 3D-printed prosthesis in terms of the bone defect size and overall distal ulna shape. The stability of the distal radioulnar joint was reconstructed by the palmaris longus tendon which was pulled tight and sutured to anchors in radius. During the final follow-up, the patients also exhibited good function in the affected limb, without evident pain, tumor recurrences, or complications such as pathologic fractures and prosthetic loosening. These results indicated that 3D-printed personalized prostheses are an effective means of bone defect reconstruction after resection of distal GCTs. As each 3D-printed prosthesis was personalized based on actual patient conditions, a suitable fit with the postresection bone defect could be achieved for the satisfaction of anatomical and biomechanical requirements.

This study has some limitations. First, this is a case report because of the low incidence of distal ulna GCTs. Therefore, further studies with a larger sample size are required to validate the benefits of our proposed treatment approach. Second, the follow-up duration was relatively short. Long-term follow-up of the patients of this study may be necessary to investigate recurrence and the survival outcomes associated with personalized prostheses. Although this study has limitations such as involving only a single patient, a short follow-up period, and the lack of comparative data, as an application of a new technology, these issues can all be improved in future work. For instance, the translational medicine concept in orthopedics, which combines 3D printing, artificial intelligence, and other new technologies with orthopedics, has accelerated the speed of theoretical transformation into clinical practice.^[28]^

In our future clinical work, we will gather more cases of distal ulna GCTs for further research.

## 4. Conclusion

A well-fitted personalized prosthesis, made using 3D printing techniques, can effectively reconstruct joint function and bone defects resulting from radical distal ulnar resection due to GCTs. This case demonstrates that the combination of radical resection of a GCT in the distal ulna and reconstruction with a 3D-printed personalized prosthesis could lead to successful oncologic and functional outcomes.

## Author contributions

**Conceptualization:** Cong Xiao.

**Data curation:** Chuanle Yang, Lijuan Liu.

**Investigation:** Chuanle Yang.

**Methodology:** Chuanle Yang, Xiaofeng Zheng.

**Resources:** Shaoyun Zhang.

**Software:** Lifu Wang.

**Supervision:** Cong Xiao.

**Writing – original draft:** Lifu Wang.

**Writing – review & editing:** Zhixiang Gao, Cong Xiao.

## Supplementary Material


