# Supplementary figures and images for: Radical resection of a giant cell tumor of the distal ulna and reconstruction with a 3D-printed distal ulnar prosthesis: A case report
Source: Medicine (Baltimore). 2025 Jul 18;104(29):e43504. doi: 10.1097/MD.0000000000043504 (PMC12282713; doi:10.1097/MD.0000000000043504)

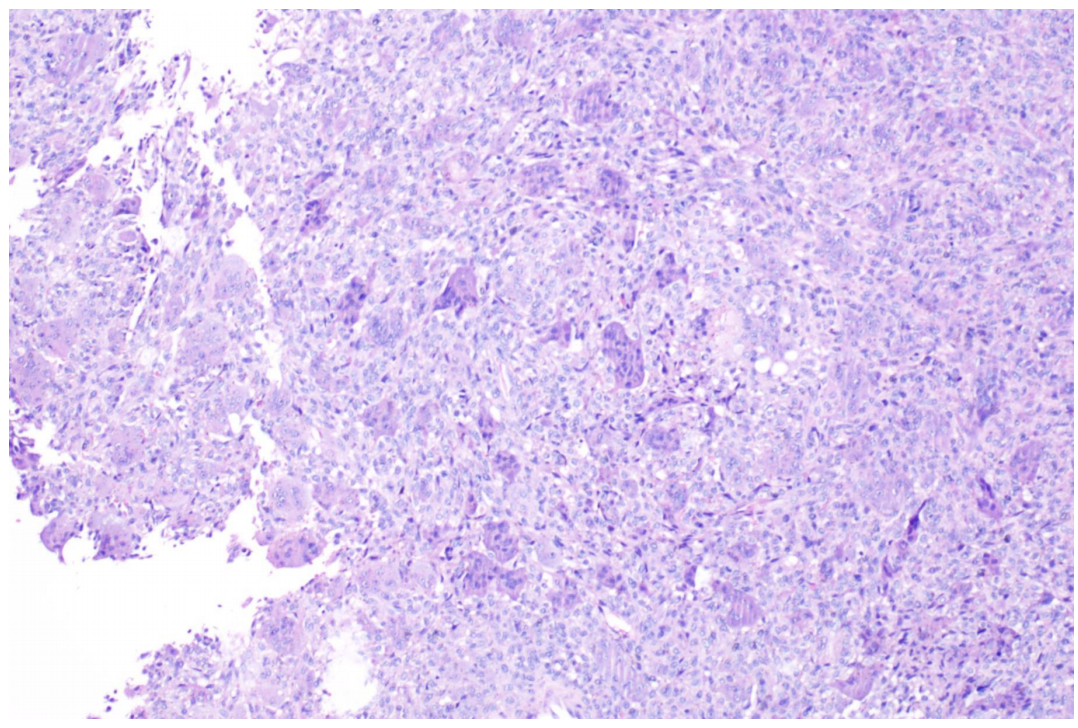

Supplement: Supplementary file 1 [file medi-104-e43504-s001.tif]
